# Characterization of Lenticulostriate Arteries and Its Associations With Vascular Risk Factors in Community-Dwelling Elderly

**DOI:** 10.3389/fnagi.2021.685571

**Published:** 2021-06-22

**Authors:** Xiaopei Xu, Xiao Wu, Chengcheng Zhu, Ruiting Zhang, Yeerfan Jiaerken, Shuyue Wang, Hui Hong, Wenke Yu, Kaicheng Li, Qingze Zeng, Xiao Luo, Xinfeng Yu, Jianzhong Sun, Minming Zhang, Peiyu Huang

**Affiliations:** ^1^Department of Radiology, The Second Affiliated Hospital, Zhejiang University School of Medicine, Hangzhou, China; ^2^Department of Radiology, University of Washington, Seattle, WA, United States

**Keywords:** lenticulostriate artery, small vessel disease, vessel morphology, aging, tortuosity

## Abstract

Lenticulostriate arteries (LSAs) supply blood to important subcortical areas and are, therefore, essential for maintaining the optimal functioning of the brain’s most metabolically active nuclei. Past studies have demonstrated the potential for quantifying the morphology of LSAs as biomarkers of vascular fragility or underlying arteriopathies. Thus, the current study aims to evaluate the morphological features of LSAs, their potential value in cerebrovascular risk stratification, and their concordance with other vascular risk factors in community-dwelling elderly people. A total of 125 community-dwelling elderly subjects who underwent a brain MRI scan were selected from our prospectively collected imaging database. The morphological measures of LSAs were calculated on the vascular skeletons obtained by manual tracing, and the number of LSAs was counted. Additionally, imaging biomarkers of small vessel disease were evaluated, and the diameters of major cerebral arteries were measured. The effects of vascular risk factors on LSA morphometry, as well as the relationship between LSA measures and other imaging biomarkers, were investigated. We found that smokers had shorter (*p* = 0.04) and straighter LSAs (*p* < 0.01) compared to nonsmokers, and the presence of hypertension is associated with less tortuous LSAs (*p* = 0.03) in community-dwelling elderly. Moreover, the middle cerebral artery diameter was positively correlated with LSA count (*r* = 0.278, *p* = 0.025) and vessel tortuosity (*r* = 0.257, *p* = 0.04). The posterior cerebral artery diameter was positively correlated with vessel tortuosity and vessel length. Considering the scarcity of noninvasive methods for measuring small artery abnormalities in the brain, the LSA morphological measures may provide valuable information to better understand cerebral small vessel degeneration during aging.

## Introduction

As one of the most critical vascular structures in the human brain, lenticulostriate arteries (LSAs) supplies blood to important subcortical areas, including the caudate nucleus, globus pallidus, putamen, and part of the posterior limb of the internal capsule (Marinković et al., 2001). Originating from the high flow middle cerebral artery (MCA), LSAs are end arteries with small outer diameters (Marinković et al., 2001). This abrupt size and flow change make LSAs especially susceptible to damage (e.g., hypertension). During aging, the arterial consequences of risk factors, such as hypertension, hyperlipidemia, diabetes, and smoking, start to surface (Chamorro et al., 1996). Thus, cerebral hypoperfusion promoted by these risk factors may eventually result in various neuropsychological diseases. Moreover, considering that perforating vessels like LSAs are essential for maintaining the optimal functioning of the brain’s most metabolically active nuclei (Wardlaw et al., 2013), *in vivo* imaging of LSAs could provide essential insights and help in understanding the physiology and mechanism of vascular events during normal aging.

Technically, LSA imaging remains challenging due to the small size of perforating arteries, and digital subtraction angiography (DSA) is considered the gold standard when visualizing smaller arteries (Kang et al., 2009b). However, due to the radioactive and invasive nature of DSA and the use of a potentially nephrotoxic contrast medium, DSA is less recommended for repeated imaging or research-oriented studies, especially in normal-aging elderly. Comparatively, high-resolution black-blood MRI offers high spatial resolution and near whole-brain coverage in a clinically acceptable time; thus, it is more suitable for imaging LSAs. Past studies have repeatedly demonstrated the potential for quantifying the morphology (e.g., branch number, length, and tortuosity) of blood vessels as biomarkers of vascular fragility or underlying arteriopathies (Taarnhøj et al., 2008; Han, 2012; Hathout and Do, 2012). For instance, the high tortuosity of the contralateral MCA was independently associated with atherosclerotic disease (Kim et al., 2015), and cervical artery tortuosity is significantly correlated with intracranial aneurysm (Labeyrie et al., 2017). However, the number of studies characterizing the morphological measurements of small arteries like LSAs is still limited.

Thus, the scope of the current study is to evaluate the morphological features of LSAs as well as their potential value in cerebrovascular risk stratification and their concordance with other vascular indexes in community-dwelling elderly. Three-dimensional high-resolution black blood MRI was used to visualize LSAs and extract geometrical measurements.

## Materials and Methods

### Participants

We searched our prospectively collected imaging database on community elderly people (age > 50) and included 125 elder subjects. Exclusion criteria include (1) history of stroke, brain trauma, neurological or psychiatric diseases, or systemic diseases that could severely affect the brain; (2) metal implants, claustrophobia, or other inappropriate conditions for MR scans; (3) existence of lacunas, microbleeds, and severe white matter hyperintensities (WMH) with Fazekas deep or periventricular score > 2, which may heavily influence perivascular space (PVS) dilation or bring bias into PVS assessment; and (4) cognitive impairment (Mini-Mental State Examination score < 24), which is likely the result of Alzheimer’s or other specific pathologies.

All subjects went through a complete assessment of neuropsychiatric conditions and multi-sequence MRI scans. Hypertension was defined as the presence of any of the following: systolic blood pressure ≥ 140 mmHg or diastolic pressure ≥ 90 mmHg twice in calm conditions or having a self-reported history of hypertension. Diabetes mellitus was defined as the presence of any of the following: fasting serum glucose >7.0 mmol/L or postprandial 2 h plasma glucose >11.1 mmol/L or having a previous history of diabetes. Hyperlipidemia was defined as having an elevated level of one of the following: triglyceride (>1.7 mmol/L), total cholesterol (>5.7 mmol/L), or low-density lipoprotein (>3.1 mmol/L).

### Image Acquisition

All the MR images were acquired using a United Imaging MR790 3.0T scanner (Shanghai, China). T1-weighted images were acquired with a 3D fast spoiled gradient-echo sequence; the parameters were as follows: TR = 6.9 ms, TE = 2.9 ms, flip angle = 9°, inversion time = 1,000 ms, field of view = 256 × 240 mm, voxel size = 1 mm × 1 mm × 1 mm, and 208 sagittal slices. T2-weighted images were acquired with a MATRIX (modulated flip angle technique in refocused imaging with extended echo train, equivalent to CUBE for GE, SPACE for Siemens, and VISTA for Philips) sequence; the parameters were as follows: TR = 3,000 ms, TE = 405.46 ms, echo train length = 180, field of view = 256 × 240 mm, voxel size = 0.8 mm × 0.8 mm × 0.8 mm, and 208 sagittal slices. T2 FLAIR images were acquired with inversion recovery MATRIX sequence; the parameters were as follows: TR = 6,500 ms, TE = 432.48 ms, echo train length = 220, bandwidth = 600 Hz/pixel, field of view = 256 × 220 mm, voxel size = 1 mm × 1 mm × 1 mm, and 170 sagittal slices. High-resolution black blood images were also acquired with a MATRIX sequence; the parameters were as follows: TR = 750 ms, TE = 23.7 ms, echo train length = 45, bandwidth = 600 Hz/pixel, field of view = 220 × 180 mm, voxel size = 0.44 mm × 0.44 mm × 0.66 mm, and 220 sagittal slices. Several other sequences were acquired, and the total scan time was about 1 h.

### LSA Visualization and Morphology Metrics

**FIGURE 1:**
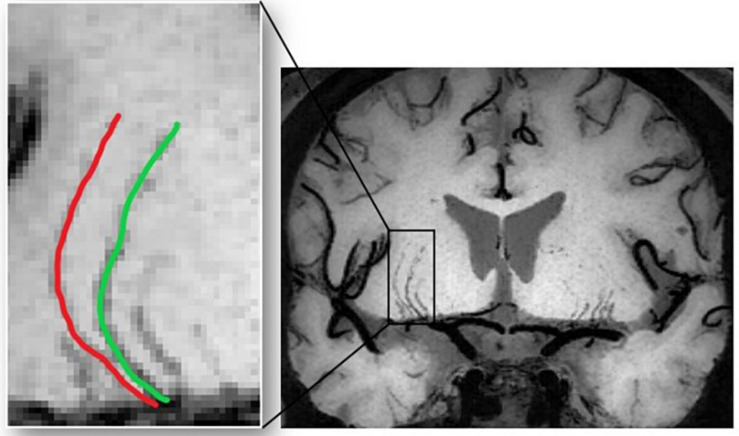
The images are coronal 10-mm thin-slice minimum intensity projections of 3D black blood MRI for presentation. LSAs were manually delineated on the images and morphological measurements were calculated accordingly.

All images were manually checked to ensure the absence of visible motion corruption or other artifacts. Three-dimensional black-blood images were consecutively reconstructed in the coronal view, created parallel to the LSAs and bilateral sagittal planes. LSAs were then visualized by thin-slab (10 mm) minimum intensity projection (minIP) and then delineated on conventional 2D minIP images. The viewer manually evaluated each slice of the minIP images and, for each LSA, chose the slice which could capture the entire length of the vessel. The minIP image was then selected to delineate the corresponding LSA. An experienced radiologist (X.W), who was blind to patient information, manually delineated the LSAs using ITK-SNAP (Yushkevich et al., 2006), as illustrated in Figure 1. After skeletonization and removal of the false spurs, each LSA segment was examined by another radiologist to ensure that the vessel skeleton truly reflected the centerline of the LSA and the delineated length was reasonable. Then we performed quantitative morphometric analysis on these data, and geometric measurements were extracted based on the skeletonized vessel.

**FIGURE 2:**
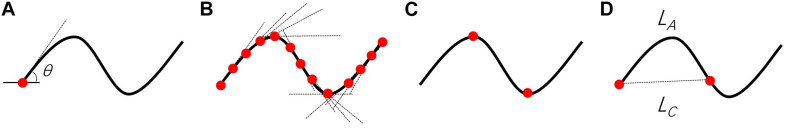
Visual demonstration of parameters required for VTI computation. **(A)** *θ* is the angle between lines tangent to each centerline pixel and the *x*-axis. **(B)** Tangent lines for points along the centerline. **(C)** *N* is the number of critical points where the first derivative of the centerline vanishes. The critical points (red circles) were determined based on changes in the sign of slope of the tangent lines. **(D)** *L*_*A*_ and *L*_*C*_ are the length of the centerline and its chord, respectively. The magnitude (*M*) of the curve is the ratio of *L*_*A*_ to *L*_*C*_ between pairs of inflection points including centerline endpoints.

The number of LSAs is defined as the LSAs that originate directly from the MCA. The entire vessel length (VL) is defined as the actual path length between the origin and the terminal of an LSA. Conventional tortuosity, or distance metric (DM), is defined as the ratio of the estimated VL to the Euclidean distance between the two endpoints of the curve. In addition, we adopted an improved quantitative vessel tortuosity index (VTI) based on the combination of several local and global centerline tortuosity features as previously explained (Khansari et al., 2017; Cano et al., 2020). Mathematically, VTI is given by the following equation:

$$\mathrm{VTI}=\frac{0.1{\text{SD}}_{\theta }.N.M.L_{A}}{L_{C}}$$

where SD_θ_ represents the local angle change, *M* is the amplitude of the curvature along the vessel centerline, *N* is the number of critical points, *L*_*A*_ is the centerline length, and *L*_*C*_ is the centerline chord length. These parameters are further demonstrated in Figure 2. Theoretically, the minimum value for VTI is zero, which corresponds to a straight line, whereas there is no theoretical maximum value for VTI.

The number of LSAs was calculated for each subject. The rest of the measures, including VL, DM, and VTI, were obtained for each LSA; subsequently, the mean, median, and standard deviation (SD) of all LSAs were summarized for each subject to quantitatively describe the vessel. All the abovementioned imaging analyses were performed using an in-house Matlab program (The MathWorks Inc., Natick, MA, United States).

### Measurement of the Artery Diameters

The diameters of the internal carotid artery (ICA) and basilar artery (BA) were measured on the axial slices of 3D-TOF MRA images by a neuroradiologist. Specifically, the ICA was measured at the vertical cavernous segment (Yeniçeri et al., 2017). The BA was measured on the slice at the middle of the pons (Ichikawa et al., 2009). The short axis was used as artery diameters to avoid the oblique effect. Diameters of the bilateral ICA were averaged for further analysis. The anterior cerebral artery (ACA), MCA, and posterior cerebral artery (PCA) were measured at 5 mm away from the bifurcation sites of the Willis’ circle, and the diameters of bilateral arteries were averaged. In order to assess intra-observer consistency, the neuro-radiologist performed second measurements on randomly selected 30 cases after 1 month. The intraclass correlation index was used to evaluate consistency.

### WMH Segmentation

White matter hyperintensities segmentation was performed using BIANCA (Brain Intensity AbNormality Classification Algorithm)^1^, which is a fully automated, supervised method for WMH detection, based on the k-nearest neighbor algorithm. We trained BIANCA with 24 subjects’ T2FLAIR images and hand depicted WMH masks. The final model included intensity information from both T1 images and T2 FLAIR images as well as the Montreal Neurological Institute spatial transformation information. We selected 2,000 training points within the WMH area and 10,000 training points from the normal-appearing white matter area. After generating the WMH probability map, we created a mask by inwardly dilating the cerebral spinal fluid (CSF) masks until the white matter was reached, using the built-in function of BIANCA. The mask was applied to the probability map, and the results were thresholded by 0.5 to derive the final WMH mask. Visual assessment and manual corrections were performed to ensure accuracy by an experienced neuroradiologist (RZ).

### Visual Assessment of Cerebral Small Vessel Disease Markers

Perivascular space dilation, lacunes, and microbleeds were visually assessed according to the STandards for ReportIng Vascular changes on nEuroimaging by an experienced neuroradiologist (RZ). As the number of observable PVS can be quite different due to slice thickness changes, to make the community cohort’s scores comparable with previous studies, we reconstructed T2 images into 5-mm axial T2W images on which the PVS was assessed. Dilated PVS was defined as a round, oval, or linear lesion smaller than 3 mm with a signal similar to that of CSF without a surrounding hyperintense rim. We used a four-point visual rating scale (0 = no PVS, 1 = ≤ 10 PVS, 2 = 11–20 PVS, 3 = 21–40 PVS, and 4 = ≥ 40 PVS) for the basal ganglia and centrum semiovale. Lacunes were defined as a round or ovoid, subcortical, fluid-filled cavity (signal similar to CSF) with diameters ranging from 3 to 15 mm, consistent with a previous acute small subcortical infarct or hemorrhage in the vicinity of one perforating arteriole. Microbleeds were defined as small, signal areas void with associated blooming seen on susceptibility-weighted MRI.

### Statistical Analysis

Sex, history of diabetes mellitus, hypertension, hyperlipidemia, and smoking were recorded as binary variables. Age, education, intracranial volume (ICV), morphological measures, and cerebral artery diameters were recorded as continuous variables. Continuous data were analyzed using the analysis of variance (ANOVA) test to examine the effects of cerebrovascular risk factors on LSA morphometry. Univariate analysis was performed using the χ^2^ test for categorical data. Spearman rank correlation analysis was used to examine the relationship between morphological measures and measures from other imaging modalities. All analyses were adjusted for age, sex, and ICV. For all the statistical analyses described above, we performed Bonferroni corrections for the problem of multiple comparisons, including ANOVA and correlation analysis. SPSS 22.0 (SPSS, Chicago, IL, United States) was used for all the statistical analyses. A significance level of *p* < 0.05 was set for all statistical tests.

## Results

### Demographics

A total of 125 subjects (mean ± SD: 59.9 ± 7.1; range: 50–82 years; and female/male: 68/57) were included. The population had an average of 7.6 years of education and had the median score for MMSE was 28, which suggested a normal cognitive function. Subjects with any of the four vascular risk factors, including hypertension, diabetes, hyperlipidemia, and smoking, account for 40.8, 15.2, 14.1, and 28.8% of the entire population, respectively. This prevalence of vascular risk factors was similar to previous community studies. We also investigated the overall cerebral small vessel disease (CSVD) burden using imaging-related CSVD biomarkers, and CSVD-related brain damages were mild, as displayed in Table 1. After quantifying the LSA number and vessel morphology for each subject, we found that the median LSA number for each subject was 8, and the median length was 17.8 mm. ICV is a common nuisance to correct for in structural MRI studies as it reflects interindividual variations in brain volume due to head size difference. We investigated the relation between ICV and LSA morphology and found that the ICV was negatively correlated with median VTI (*p* = 0.01). We then studied the association between LSA morphology measurements and vascular risk factors. Only the significant findings will be described below unless otherwise stated.

**TABLE 1:** Subjects demographics.

	*N* = 125
**Demographic characteristics**	
Age, y, mean (sd)	59.9 (7.1)
Sex, f/m	68/57
Education, y, mean (sd)	7.6 (4.0)
**Vascular risk factors, *n* (%)**	
Hypertension	51 (40.8%)
Diabetes	19 (15.2%)
Hyperlipidemia	18 (14.4%)
Smoking	36 (28.8%)
**Cognitive assessments, median (interquartile range)**	
MMSE score	28 (26∼29)
**CSVD imaging markers, median (interquartile range)**	
WMH volume, ml	1.5 (0.8∼2.8)
Deep white matter WMH score	1 (0∼1)
Peri-ventricular WMH score	1 (1∼1)
Deep white matter PVS score	1 (1∼2)
Basal ganglia PVS score	1 (1∼1)
Lacune, *n*	0 (0∼0)
Microbleeds, *n*	0 (0∼0)
**LSA features, median (interquartile range)**	
Number	8 (7∼9)
Length, mm	17.8 (16.0∼20.5)

*Sd, standard deviation; f, female; m, male; MMSE, Mini-Mental State Examination; CSVD, cerebral small vessel disease; WMH, white matter hyperintensity; and LSA, lenticulostriate artery.*

### Demographics and Risk Factors’ Effect

**FIGURE 3:**
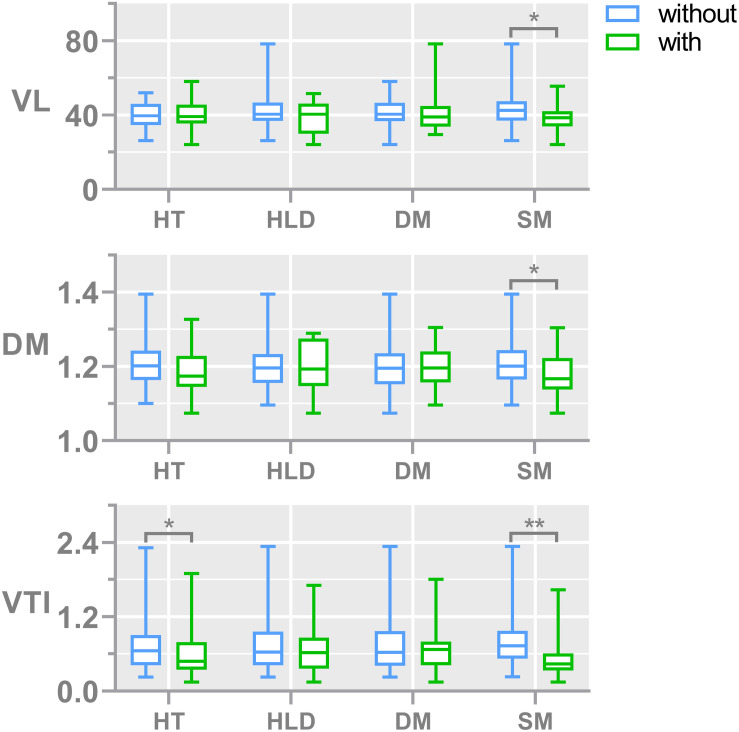
Bar plots show the comparisons of vessel morphological measures, including the median of DM, VTI, and VL, between subjects with and without different risk factors. The numbers of subjects with and without hypertension are 51 and 74, respectively; the numbers of subjects with and without diabetes are 19 and 106, respectively; the numbers of subjects with and without hyperlipidemia are 18 and 107, respectively; the numbers of subjects with and without smoking are 36 and 89, respectively. The median number of LSA counts in all subjects was 8. DM, median distance metric; VTI, median vessel tortuosity index; VL, median vessel length; HT, hypertension; HLD, hyperlipidemia; DIA, diabetes mellitus; and SM, smoking.

During the aging process, the age was associated with reduced median VL (weak association, *r* = -0.165, *p* = 0.065). The relationship between vascular risk factors and LSA measures is shown in Figure 3. Subjects with hypertension generally had lower median VTI than non-hypertensive subjects (*p* = 0.03). Smokers tended to have straighter and shorter LSAs compared to nonsmokers as we found significantly lower median DM (*p* = 0.01), lower median VTI (*p* < 0.01), and lower median VL (*p* = 0.04) in subjects with smoking history. There was no association between LSA count and the aforementioned risk factors. None of the CSVD markers were associated with morphological vessel features.

### Relationship Between Vessel Morphology and Other Imaging Features

**FIGURE 4:**
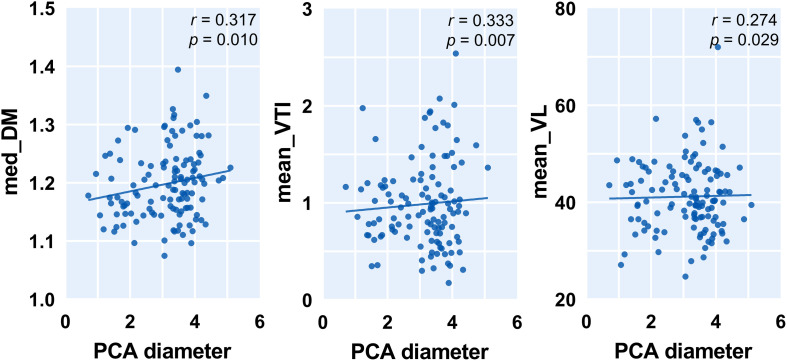
Correlation between posterior cerebral artery (PCA) diameter and measurements of lenticulostriate arteries (LSAs). Med, medium; DM, distance metric; VTI, vessel tortuosity index; and VL, vessel length.

**FIGURE 5:**
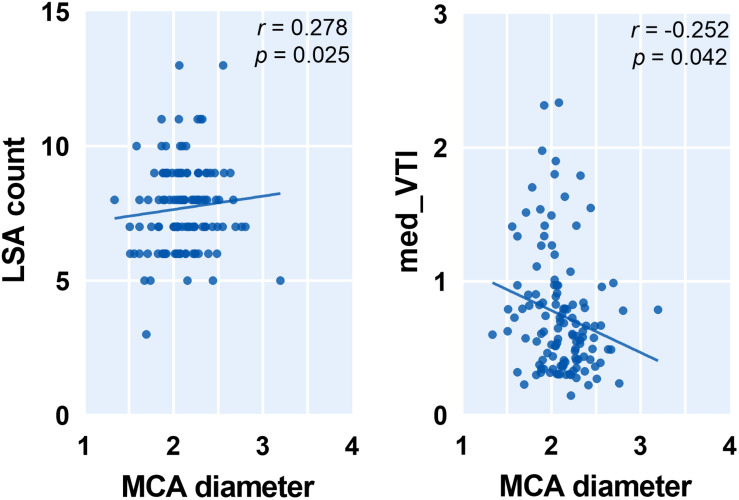
Correlation between middle cerebral artery (MCA) diameter and measurements of lenticulostriate arteries (LSAs). Med, medium; VTI, vessel tortuosity index.

To capture the relation between major large vessels of the brain and LSA morphology, we then studied the relationship between vessel diameter and LSA measures. Interestingly, the cerebral artery which was most relevant to LSA morphology measurements is the PCA. We found that a wider PCA was associated with longer and more tortuous LSAs, demonstrated by a higher PCA diameter which was correlated with significantly higher median DM (*r* = 0.317, *p* = 0.010), higher mean VTI (*r* = 0.333, *p* = 0.007), higher mean VL (*r* = 0.274, *p* = 0.029), and higher median VL (*r* = 0.306, *p* = 0.014). Details are shown in Figure 4. Moreover, the MCA diameter was correlated positively with LSA count (Figure 5, *r* = 0.278, *p* = 0.025) and negatively with median VTI (*r* = −0.252, *p* = 0.042).

## Discussion

Lenticulostriate arteries branching from MCA are among the most critical perforating arteries in the human brain and the sites of many neurologic diseases. Considering that LSAs are the sole blood supply of basal ganglia and its vicinity in the brain, the perfusion status of these essential subcortical nuclei is thus primarily dependent on the “wellbeing” of LSAs. As such, the characterization of LSAs may provide crucial information on risk stratification. Past studies showed that LSA stems and branches had been correlated with hypertension, a major risk factor for cerebrovascular diseases (Kang et al., 2009a). A large population study based on DSA also demonstrated significantly lower LSA counts in patients with CSVD than normal subjects (Chen et al., 2016). This evidence suggested that increased cerebrovascular risk factors might associate with abnormal or deformed LSA configuration. However, the physical cause underlying and governing the shape of normal and abnormal vessels might be completely different. Before quantifying the phenomenon of abnormal tortuosity, some understanding of the shape of the normal, or physiologic, tortuosity is required. In the current study, we were not able to establish the connection between LSA counts and cerebrovascular risk factors; this might relate to the fact that all of our study subjects were community-dwelling elderly with a lower level of baseline risk factors compared to previous studies (Kang et al., 2009a; Chen et al., 2016). Nevertheless, we still found that the LSA length is shorter in smokers compared to nonsmokers. Okuchi et al. (2013) had a similar observation that subjects with smoking histories had a shorter total length of LSA branches, although the difference is not statistically significant. Thus, to amplify the effects of cerebrovascular risk factors during normal aging, we used morphological measurements that are more comprehensive than conventional semiquantitative measurements like LSA count to characterize LSA structure change.

Arterial tortuosity, or alternatively stated, the presence of abnormal twists of arteries, is known to associate with older age, female sex, and many cardiovascular factors (Del Corso et al., 1998; Cha et al., 2003). This is in accordance with our results which showed that female subjects have more tortuous LSAs. As a marker of vascular fragility or a useful indicator of underlying arteriopathies, tortuosity has considerable clinical potential and research utility in the baseline stratification of various vascular diseases. Pancera et al. (1998, 2000) presented an association between arterial hypertension and kinking of the carotid artery assessed by Echo-Doppler in two cross-sectional studies. Hiroki et al. (2002) also found that the tortuosity of the white matter medullary arterioles is related to the severity of hypertension. However, this correlation is still a matter of controversy. An opposite relation, namely, decreasing retinal arterial tortuosity with increasing blood pressure, was found in healthy subjects (Taarnhøj et al., 2008), which is in accordance with our results that hypertension is associated with less tortuous LSAs. Notably, another study (Witt et al., 2006) found that ischemic heart disease was associated with reduced retinal arterial tortuosity, independent of arterial blood pressure. This is parallel and supports the finding that in the present study, increasing hypertension, a risk factor for IHD, was also associated with decreasing LSA tortuosity. This inconsistency among different studies may be due to limitations in the measuring method, these morphological differences, or genetic factors. When analyzing the normal population, there might be a wide range of genetic variability across different subjects, making genetic factors pose a more significant effect on aging and risk factors. We also speculate that due to the microscopic anatomy, different types or sites of the arteries might respond differently to certain risk factors. For instance, the tortuosity of large or muscular arteries might be positively associated with the presence of hypertension, whereas the relationship has reversed between small arteries and hypertension. Future work is needed to determine if the different populations or different arterial types have a specific influence on vessel tortuosity.

Moreover, to the best of our knowledge, we are the first to demonstrate the relationship between LSA morphology and the diameters of cerebral arteries. We found that patients with fewer LSAs tend to have narrower ACA and MCA. The reasonable explanation behind this might be that subjects with narrower cerebral arteries are less likely to have an optimal cerebral perfusion status; thus, only fewer perforating arteries like LSAs could be observed. In addition, we found that subjects with longer and more tortuous LSAs usually had wider PCA. Clinical and experimental studies have demonstrated a strong association between vessel tortuosity and mechanical factors such as blood pressure, blood flow, axial tension, and structural wall changes (Han, 2012). Although the temporal order between the decrease in vascular tortuosity of LSAs and PCA narrowing is not clear, we hypothesize that vascular tortuosity influences hemodynamics and altered hemodynamics are associated with vascular remodeling. It is a well-known fact that compliance of the central arteries effectively dampens hemodynamic pulsations to deliver highly continuous flow to the cerebral microcirculation. Thus, reduced arterial wall distensibility, as measured by decreased PCA diameter, might be responsible for the remodeling of bifurcation. Our speculation is in accordance with a previous study that showed that increased vessel tortuosity and reduced vascular compliance of LSAs were associated with aging (Schnerr et al., 2017). With the hardening of the arterial wall and increased resistance of the cerebral vasculature, the pulsatility in the LSA and intracerebral damping of the blood pulse wave from the MCA to the LSA were significantly lower in the older subjects (Schnerr et al., 2017). Interestingly, the morphological measurements of LSAs were associated with the diameters of both MCA, which is the parent artery of LSAs, and nonparent arteries like ACA and PCA. This suggested that an abnormal vasculature system increases the geometric resistance to blood flow, decreases overall perfusion, and, in general, leads to these microvascular shape abnormalities (Jain, 2001). It should be noted that we only used the short-axis diameter of the artery to avoid potential bias caused by measurements obtained from planes non-perpendicular to the longitudinal axis of the artery (Ma et al., 2019).

There are several limitations to our study. First, visualization and quantification of vessel morphometry were performed on a 2D projection image and was, thus, less capable of analyzing arteries that are course perpendicular to the coronal plane. Although using 3D geometric models might yield more comprehensive results, these methods are still in an investigational stage. Thus, we chose a more straightforward method that can be easily applied in clinical practice. Second, our cross-sectional study does not firmly demonstrate the causality of vascular tortuosity. Future prospective, large-scale follow-up studies using a normal population are needed to determine causality and underlying pathophysiology. Last, previous studies reported that vascular tortuosity differs according to ethnicity (Masuda et al., 2004), as certain genetic mutations are known to affect arterial tortuosity (Faiyaz-Ul-Haque et al., 2009). Thus, differences in genetic variability may contribute to the different degrees of tortuosity of the cerebral arteries and different locations of atherosclerosis in an individual patient. Further studies are required to explore this hypothesis.

## Conclusion

In the current study, we applied 3D high-resolution black blood MRI to visualize LSAs and quantify their morphological features in community-dwelling elderly people. As a result, we successfully demonstrated that the morphological features of LSAs are associated with cerebrovascular risk factors. Moreover, less tortuous LSAs are associated with narrower cerebral arteries measured by vessel diameters. Considering the scarcity of noninvasive methods for measuring cerebral small artery abnormalities, the LSA morphological measures may provide valuable information to elucidate cerebral small vessel degeneration during aging.

## Data Availability Statement

Data are available upon reasonable request. Requests to access the datasets should be directed to MZ, zhangminming@zju.edu.cn.

## Ethics Statement

The studies involving human participants were reviewed and approved by the Medical Ethic Committee of The Second Affiliated Hospital of Zhejiang University School of Medicine. The patients/participants provided their written informed consent to participate in this study.

## Author Contributions

XX and XW conducted the investigation and wrote the main manuscript. RZ, YJ, SW, HH, WY, KL, QZ, XL, XY, and JS facilitated patient recruitment data acquisition and literature retrieval. CZ, MZ, and PH designed and organized the study, and provided critical revisions to the manuscript. All authors contributed to the article and approved the submitted version.

## Conflict of Interest

The authors declare that the research was conducted in the absence of any commercial or financial relationships that could be construed as a potential conflict of interest.

## Footnotes

1 https://fsl.fmrib.ox.ac.uk/fsl/fslwiki/BIANCA
